# Noble Gas in a Ring

**DOI:** 10.3390/molecules26154677

**Published:** 2021-08-02

**Authors:** Wei-Te Lin, Ya-Jyun Shih, Tzu-Jeng Hsu, Wei-Ping Hu

**Affiliations:** 1 Department of Chemistry and Biochemistry, National Chung Cheng University, Chia-Yi 621, Taiwan; wadelin1020@gmail.com (W.-T.L.); yajiun610@gmail.com (Y.-J.S.); 2 Department of Mechanical Engineering, Chung Yuan Christian University, Chung-Li 320, Taiwan; tjhsu10@gmail.com

**Keywords:** noble-gas chemistry, cyclic molecules, stability of noble-gas molecules, bonding of noble gas

## Abstract

We have designed a new type of molecule with a noble gas (Ng = Kr and Xe) atom in a six-membered ring. Their structures and stability have been studied by density functional theory and by correlated electronic structure calculations. The results showed that the six-membered ring is planar with very short Ng–O and Ng–N polar covalent bonds. The calculated energy barriers for all the unimolecular dissociation pathways are higher than 20 and 35 kcal/mol for Ng = Kr and Xe, respectively. The current study suggests that these molecules and their derivatives might be synthesized and observable at cryogenic conditions.

## 1. Introduction

**Figure 1:**
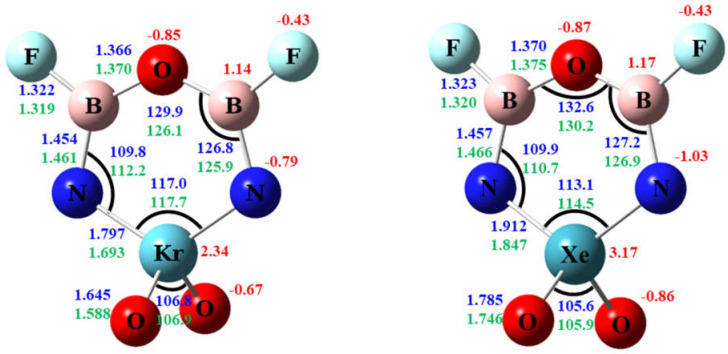
Calculated structure of NgO_3_N_2_B_2_F_2_ (Ng = Kr, Xe). The bond distances are in angstroms and the bond angles in degrees. The numbers in blue and green are values calculated by the B3LYP/aptz and MP2/aptz methods, respectively. The values in red are NBO atomic charges at B3LYP/aptz level.

Recent developments in noble gas chemistry have shown that noble gases can participate in various types of chemical bonding, such as in the molecules HNgF [1,2,3,4], HNgCN [5,6], HNgCCH [7,8,9], FNgCCH [10,11], FNgBNH [12], NgAuF [13,14], FNgO^−^ [15], FNgCC^−^ [16], FNgBN^−^ [17], CH_3_OHXeF^+^ [18], CH_3_CNXeF^+^ [19], etc. Studies in the last two decades suggest that, except for neon, all other noble gas atoms can form kinetically stable neutral molecules or anions at cryogenic conditions. However, due to the very limited varieties of chemical groups that can bond to noble gas atoms and the low bonding energies, it is difficult to extend the noble-gas containing molecules to larger or cyclic molecules except for pure electrostatic association between a very electropositive site and a noble gas atom [20]. In stable noble-gas containing molecules of the type X–Ng–Y, the noble gas atom usually bonds to H and F atoms, or chemical groups such as CC, CN and BN, which are either univalent or linear in bonding direction, and it is thus difficult to form a ring. In an earlier study of NXeO_3_^−^ [21] and related molecules, we have shown that Xe and N can form strong bonding when the Xe atom is bonded to multiple oxygen atoms in the same molecule. The O_2_Xe–N bonding can thus provide the necessary bond energy and suitable bond angle to form a potentially stable cyclic noble-gas molecule. In the current study, we also exploit the flexible –B–O–B–motif [22] to build a cyclic noble-gas containing molecule NgO_3_N_2_B_2_F_2_ (Ng = Kr and Xe) ring as shown in Figure 1. To our knowledge, this is the first study of molecules with noble gas atom in a six-membered ring. The structures, stability, and electron density distribution of the cyclic molecules will be investigated.

## 2. Methods

Molecular structures and vibrational frequencies were calculated using B3LYP [23] hybrid functional and MP2 [24] theory with the aug-cc-pVTZ [25,26] basis set. The B3LYP functional used with a large basis set has been shown to be reasonably accurate both on the bond energies and structures for noble-gas containing molecules [19,27,28,29]. For Xe, the aug-cc-pVTZ-pp basis set was used where the 28 core electrons were represented by a relativistic effective potential [30] which takes the scalar relativistic effects into account. Diffuse functions were included because recent research shows they are crucial for obtaining accurate bond energies for noble-gas containing molecules [31]. The basis set is abbreviated as aptz in the rest of this article. The intrinsic reaction coordinate (IRC) was calculated for every transition states located. The M06-2X [32,33] functional with the same basis set was used to obtain better energetics along unimolecular dissociation pathways. Coupled-cluster CCSD(T) [34] energies were calculated at B3LYP/aptz geometry with the aptz basis set to take the high-level correlation effects into account. The electron density distribution was obtained using the B3LYP/aptz method. Topology analysis [35] of the electron density was carried out using the Multiwfn program [36]. The electronic structure calculation was performed using the Gaussian 16 program, revision C01 [37].

## 3. Results and Discussion

### 3.1. Structure

The calculated structures of the ring molecules for Ng = Kr and Xe at B3LYP/aptz and MP2/aptz level are shown in Figure 1. The six-membered ring and the two fluorine atoms are coplanar, and the NgO_2_ plane is perpendicular to the ring. The structures are in C*2v* symmetry. The major differences of the two structures are on the Ng–N and Ng–O bond distances. At the B3LYP/aptz level, the Kr–N and Xe–N distances are 1.797 Å and 1.912 Å, respectively, and the Kr–O and Xe–O distances are 1.645 Å and 1.785 Å, respectively. At the MP2/aptz level, the calculated Ng–N distances are 0.07–0.10 Å shorter, and the Ng–O distances are 0.04–0.06 Å shorter. These bonds are short compared with earlier studies [21,38,39,40] and can be assigned as double bonds. All other structural parameters are similar for Ng = Kr and Xe, with corresponding bond lengths within 0.005 Å and bond angles within 4 degrees at both theoretical levels. All calculated structures are included in the Supplementary Materials.

### 3.2. Stability

We consider three unimolecular dissociation pathways of the ring molecules:

NgO_3_N_2_B_2_F_2_ (**R**) → NgO_2_N_2_B_2_F_2_ (**C1**) + O → NgON_2_B_2_F_2_ (**P1**) + O_2_ (1)

NgO_3_N_2_B_2_F_2_ (**R**) → NgO_2_ + ON_2_B_2_F_2_ (**P2**) (2)

NgO_3_N_2_B_2_F_2_ (**R**) → NgON_2_B_2_F_2_ (**P1**) + O_2_ (3)

**Figure 2:**
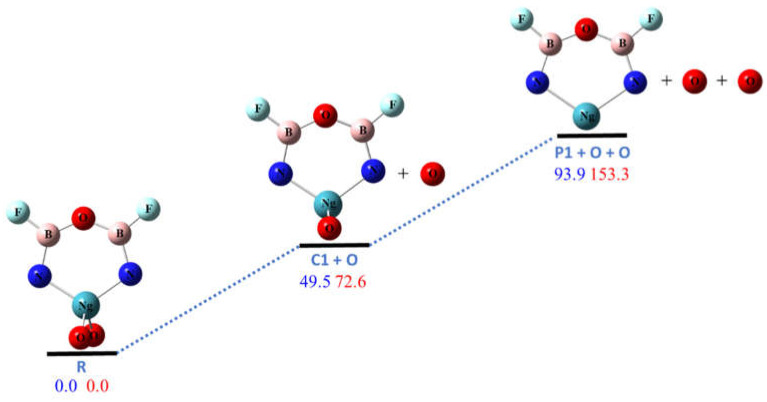
The calculated potential energy (in kcal/mol) profile for the dissociation pathway R1 at CCSD(T)/aptz//B3LYP/aptz level. The values in blue are for Ng = Kr and values in red are for Ng = Xe. The oxygen atoms are assumed to be in singlet state.

**Figure 3:**
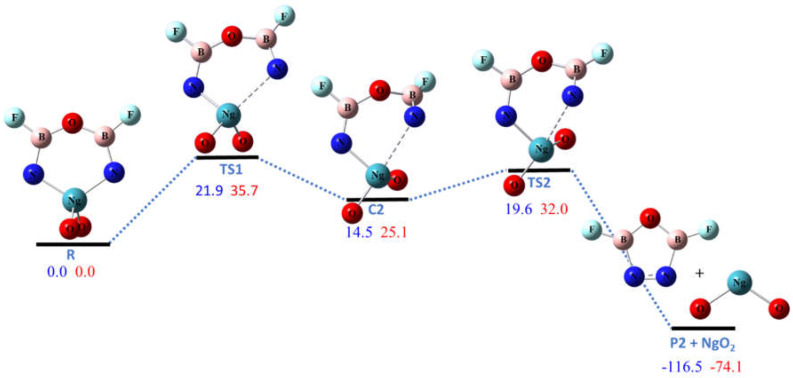
The calculated potential energy (in kcal/mol) profile for the dissociation pathway R2 at CCSD(T)/aptz//B3LYP/aptz level. The values in blue are for Ng = Kr and values in red are for Ng = Xe.

**Figure 4:**
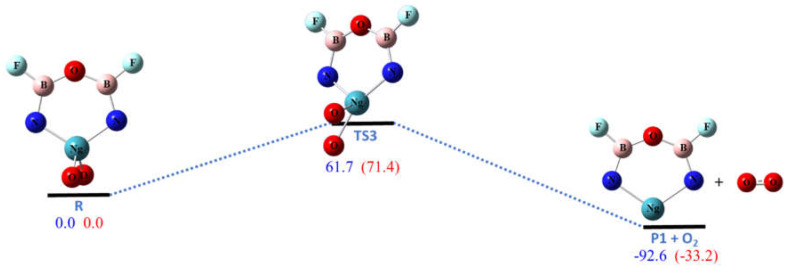
The calculated potential energy (in kcal/mol) profile for the dissociation pathway R3 at CCSD(T)/aptz//B3LYP/aptz level. The values in blue are for Ng = Kr and values in red are for Ng = Xe. The oxygen molecule is assumed to be in singlet state.

**Figure 5:**
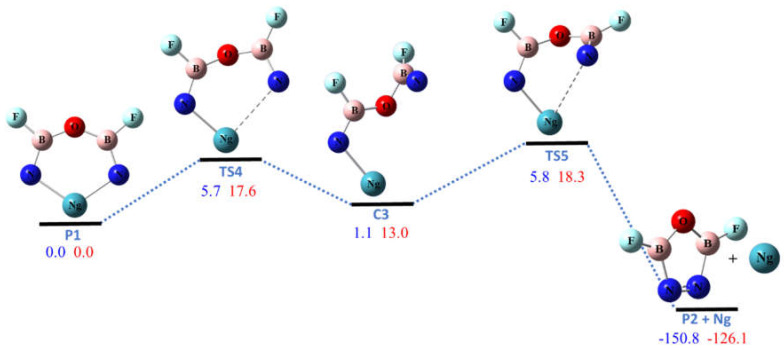
The potential energy (in kcal/mol) profile for the dissociation of NgON_2_B_2_F_2_ (Ng = Kr, Xe) at CCSD(T)/aptz//B3LYP/aptz level. The values in blue are for Ng = Kr and in red are for Ng = Xe.

The calculated potential energy profiles along these pathways are shown in Figure 2, Figure 3, Figure 4 and Figure 5. Pathway R1 is the sequential dissociation of the two oxygen atoms (with the intermediate **C1**) that are bonded to the noble gas atom. As shown in Figure 2, both steps need significant amount of energy (with the product oxygen atoms in the singlet state). For Ng = Kr the first dissociation requires ~50 kcal/mol, and the second requires ~45 kcal/mol. For Ng = Xe the first dissociation requires ~73 kcal/mol, and the second requires ~80 kcal/mol. As shown in the Supplementary Materials, the three theoretical levels, B3LYP, M06-2X, and CCSD(T), give consistent results. The MP2 results, however, may have somewhat overestimated the stability [10,15,16,21,29]. It is noted that the ground state of oxygen atom is triplet, which is ~2 eV lower than the singlet state. Dissociation to triplet oxygen atoms is spin- forbidden but could occur through intersystem crossing. We estimated the singlet-triplet crossing points to be 26 and 38 kcal/mol higher than R for Ng = Kr and Xe respectively. We performed a relaxed energy scan along the oxygen dissociation coordinates. However, we could not find any transition states for R1. These dissociation pathways seem to be barrierless. We will discuss the CCSD(T) relative energies in the rest of this article. As shown in Figure 2, the final product **P1** is also a molecule with a noble gas atom in a six-membered ring (NgON_2_B_2_F_2_) but with longer (by 0.2–0.3 Å) Ng–N bond distances. The stability of this molecule will be discussed later in this section.

Pathway R2 is the dissociation of NgO_2_ molecule to form the five-membered ring **P2** molecule (ON_2_B_2_F_2_). As shown in Figure 3, this pathway is highly exoergic due to the formation of the N–N bond. Since the Xe–N bonds are stronger than Kr–N bonds in NgO_3_N_2_B_2_F_2_, the energy of reaction of R2 for Ng = Xe is ~40 kcal/mol higher than that for Ng = Kr. The pathway was predicted to be a two-step process, with the energy barrier of the first (ring-opening) step slightly higher than that of the second (ring-closure) step. There is an intermediate complex **C2** connecting the two steps with energies of 15 and 25 kcal/mol higher than NgO_3_N_2_B_2_F_2_ for Ng = Kr and Xe, respectively. The ring-opening barriers for Ng = Kr and Xe are 21.9 and 35.7 kcal/mol, respectively, which are high enough to make the ring molecules **R** kinetically stable against dissociation at cryogenic condition. 

Pathway R3 is the one-step dissociation of O_2_ molecule to form the same six-membered ring molecule **P1** as in R1. The energies of reactions of R3 are lower than those of R1 by the bond energy of singlet O_2_ molecule. As shown in Figure 4, the calculated barriers for Ng = Kr and Xe are 61.7 and 71.4 kcal/mol, respectively, which are also high enough to make the ring molecules **R** kinetically stable at low temperature. 

The six-membered ring molecule KrON_2_B_2_F_2_, which is the product of R1 and R3, was found to be unstable against the two-step dissociation to the noble-gas atom and the **P2** molecule, as shown in Figure 5, with barriers only ~6 kcal/mol. For XeON_2_B_2_F_2_ the dissociation barriers were predicted ~18 kcal/mol, which seems still high enough to make them kinetically stable at low temperature. We did not find other low-energy unimolecular dissociation pathways for **R** and **P1**. The singlet-triplet (S-T) energy gaps for the ring molecules **R** were calculated to be higher than 70 kcal/mol at the structures in Figure 1. This indicates they are not susceptible to dissociation by intersystem crossing. However, the S-T gap for XeON_2_B_2_F_2_ (**P1**) was found to be only 37 kcal/mol due to the much longer Xe–N bonds. Thus the XeON_2_B_2_F_2_ molecule is much more susceptible to bond dissociation via intersystem crossing. The calculated structural parameters of transition states (**TS1**–**TS5**), dissociation complexes (**C1** and **C2**) and products (**P1** and **P2**) are listed in the Supplementary Materials. As shown in the Supplementary Materials, in most cases the dissociation barriers predicted at M06-2X level are in good agreement to those at CCSD(T) level. This suggests that the stability against unimolecular dissociation of the cyclic noble-gas containing molecules **R** can be modeled accurately using the M06-2X functional at only a fraction of the cost of CCSD(T) theory. The B3LYP functional predicts somewhat lower barriers while the MP2 theory predicts significantly higher barriers. 

### 3.3. Charge Distribution & Electron Density

**Figure 6:**
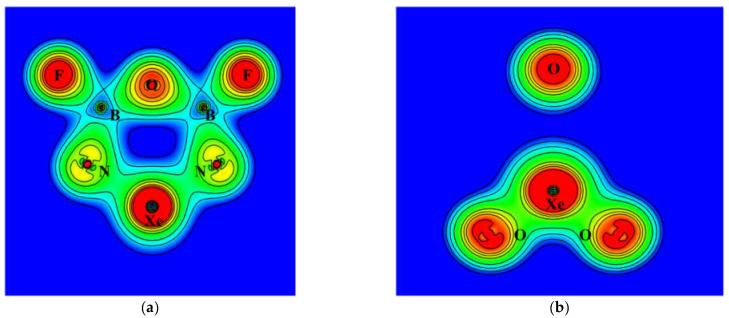
Contour plots of the calculated electron density of XeO_3_N_2_B_2_F_2_ on (**a**) the ring plane (**b**) the XeO_3_ plane.

Figure 1 shows the calculated NBO atomic charges of **R** based on the B3LYP density. The noble gas atoms were assigned very positive charges of 2.3 and 3.2 for Kr and Xe, respectively, while the oxygen and nitrogen atoms were assigned very negative charges. As shown in the figure, the charge separation is more pronounced for Ng = Xe. The contour plots of electron density of **R** (Ng = Xe) are shown in Figure 6. On the plane of the six-membered ring, the electron density distribution is consistent with polar Xe–N bonds and nearly ionic centers of boron atoms surrounded by nitrogen, fluorine, and oxygen atoms. The center of the ring is devoid of electron density. 

**Figure 7:**
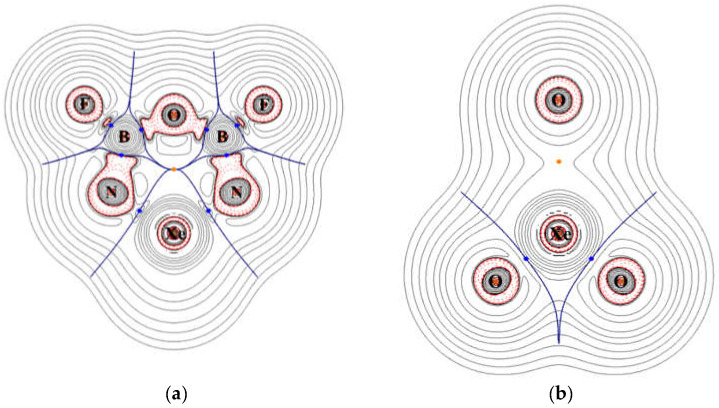
Contour plots of the calculated Laplace concentration of XeO_3_N_2_B_2_F_2_ on (**a**) the ring plane (**b**) the XeO_3_ plane. The red contour lines are in regions of charge concentration and the black contour lines are in regions of charge depletion. The blue lines pass points of zero gradients, and the dots are bond critical points.

On the plane of XeO_3_, which is perpendicular to the ring plane, the density distribution shows polar Xe–O bonds and an isolated oxygen atom that is separated from the Xe atom by the central void. The contour plots of the Laplace concentration of electron and the topology analysis are shown in Figure 7. The figure shows that the regions between Xe–N and Xe–O bonds are of electron density depletion, which indicates more ionic character. The ∇^2^*ρ* calculated at the bond critical points of Xe–N and Xe–O are 0.178 and 0.217, respectively. According to previous studies [41,42,43], the positive values suggest ionic characters. As shown in Figure 7, the critical points of B–N, B–F, and B–O are located right on the borders between regions of electron depletion and electron concentration, and the signs of the ∇^2^*ρ* are less meaningful. Thus, even though their ∇^2^*ρ* values are all positive, these bonds are more appropriately assigned as polar covalent. The detailed results of topology analysis are listed in the Supplementary Materials. The electron density contour and Laplace concentration plots of **P1** is also included in the Supplementary Materials.

## 4. Conclusions

**Figure 8:**
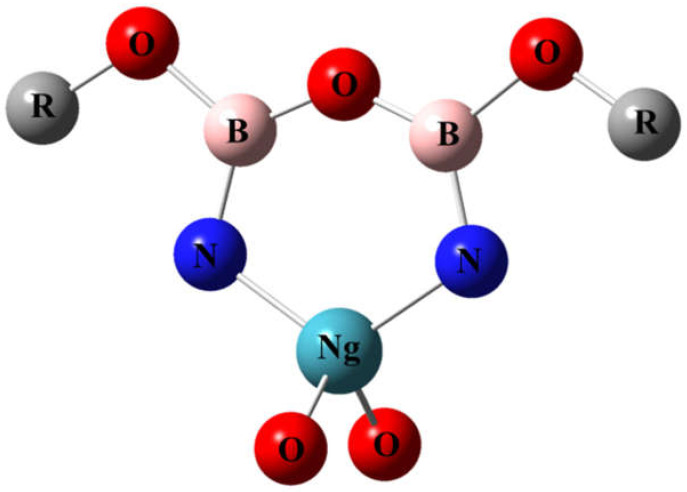
Possible derivatives of NgO_3_N_2_B_2_F_2_.

We have designed a new type of molecule with a noble-gas atom in a planar six-membered ring, which have not been studied before. High-level theoretical calculation suggests that the NgO_3_N_2_B_2_F_2_ molecules (Ng = Kr and Xe) are kinetically stable against unimolecular dissociation reactions. One may also imagine that if the fluorine atoms are replaced with oxygen atoms, as shown in Figure 8, other functional groups may be attached to the oxygen atoms to form a series of derivatives. It is anticipated that these molecules and derivatives could be observed in future experiments at cryogenic conditions.

## Data Availability

Not applicable.

## Supplementary Materials

The following are available online. Figures and tables of calculated structures, tables of the calculated relative energies and vertical singlet-triplet gaps by various theoretical methods. Figures of electron density and Laplace concentration.
