# Supplementary material for: Noble Gas in a Ring
Source: Molecules. 2021 Aug 2;26(15):4677. doi: 10.3390/molecules26154677 (PMC8347956; doi:10.3390/molecules26154677)
Supplement: Supplementary file 1 [file molecules-26-04677-s001.zip › molecules-1241967-supplementary.pdf]

Supporting Information for:

Noble Gas in a Ring

Wei-Te Lin[a], Ya-Jyun Shih[a], Tzu-Jeng Hsu[b], and Wei-Ping Hu[a],*

[a]Department of Chemistry and Biochemistry, National Chung Cheng University, Chia-Yi, Taiwan, R.O.C.

[b]Department of Mechanical Engineering, Chung Yuan Christian University, Chung-Li, Taiwan, R.O.C.

Correspondence: W.-P. Hu* (chewph@ccu.edu.tw)

26 Pages, 7 Figures, and 12 Tables

Submitted to *Molecules*, July 2021

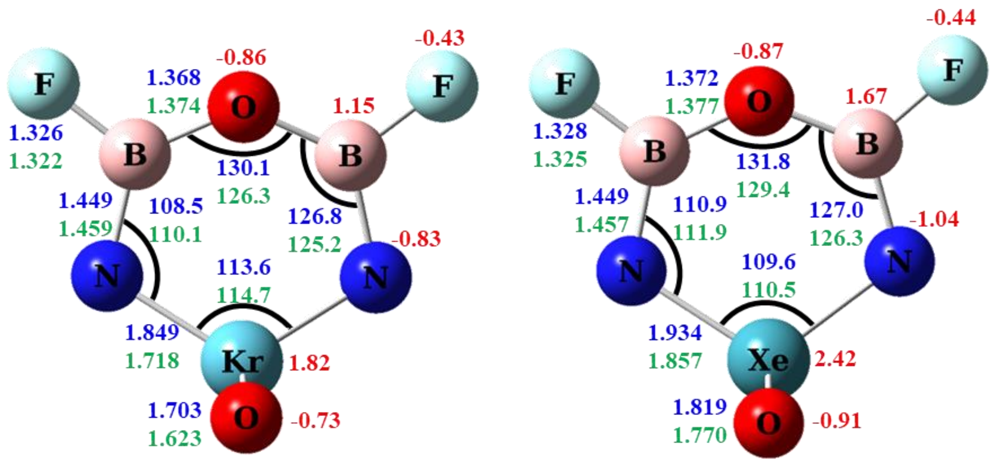

Figure S1. Calculated structure of **C1**, $NgO_2N_2B_2F_2$ (Ng = Kr, Xe) at B3LYP/aptz and MP2/aptz levels. The numbers in blue and green are values calculated by the B3LYP/aptz and MP2/aptz methods, respectively. The bond distances are in angstroms and the bond angles in degrees. The values in red are NBO atomic charges calculated at B3LYP/aptz level.

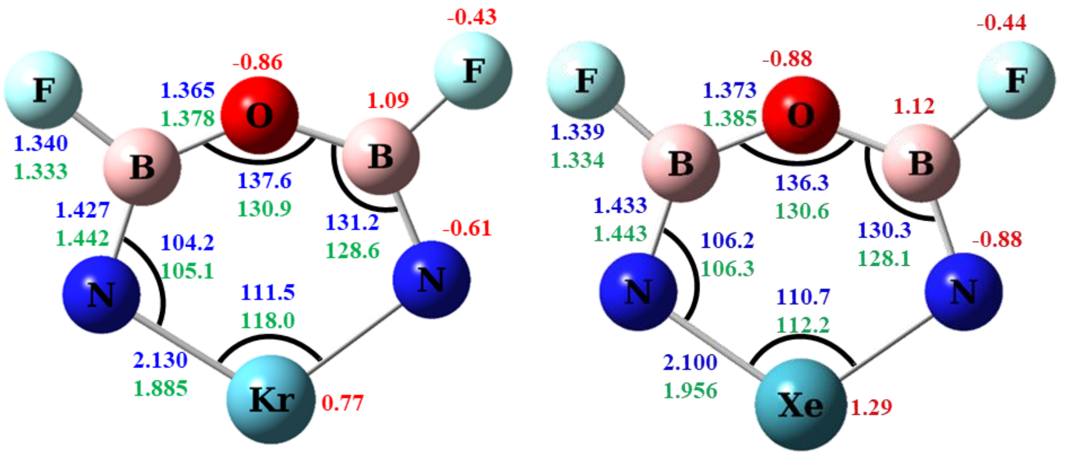

Figure S2. Calculated structure of **P1**, $NgON_2B_2F_2$ (Ng = Kr, Xe) at B3LYP/aptz and MP2/aptz levels. The bond distances are in angstroms and the bond angles in degrees. The numbers in blue and green are values calculated by the B3LYP/aptz and MP2/aptz methods, respectively. The values in red are NBO atomic charges calculated at B3LYP/aptz level.

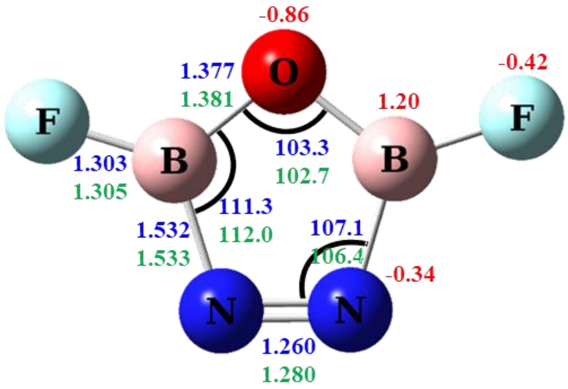

Figure S3. Calculated structure of **P2**, $ON_2B_2F_2$ at B3LYP/aptz and MP2/aptz levels. The numbers in blue and green are values calculated by the B3LYP/aptz and MP2/aptz methods, respectively. The bond distances are in angstroms and the bond angles in degrees. The values in red are NBO atomic charges calculated at B3LYP/aptz level.

(a). 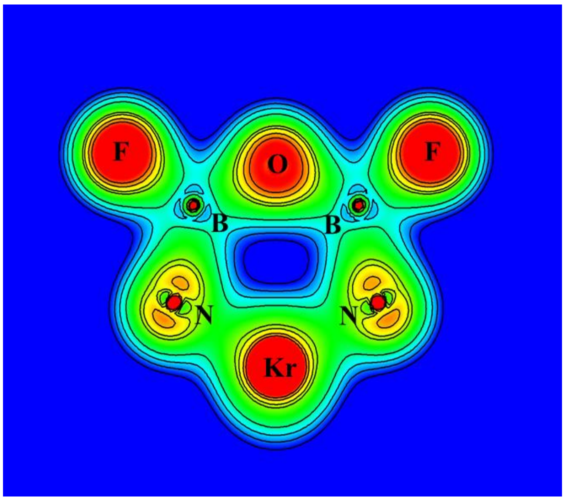

(b). 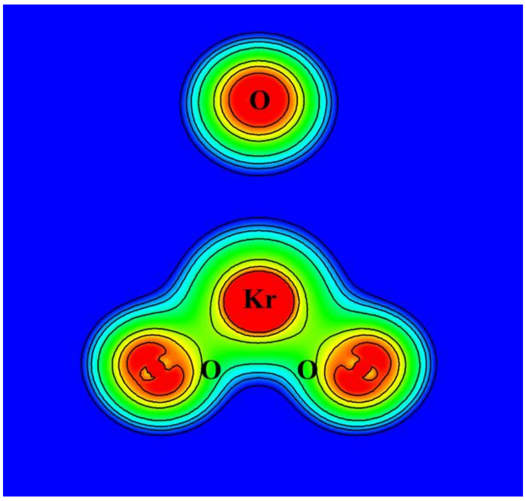

Figure S4. Contour plots of the calculated electron density of $KrO_3N_2B_2F_2$ on (a) the ring plane (b) the KrO_3 plane.

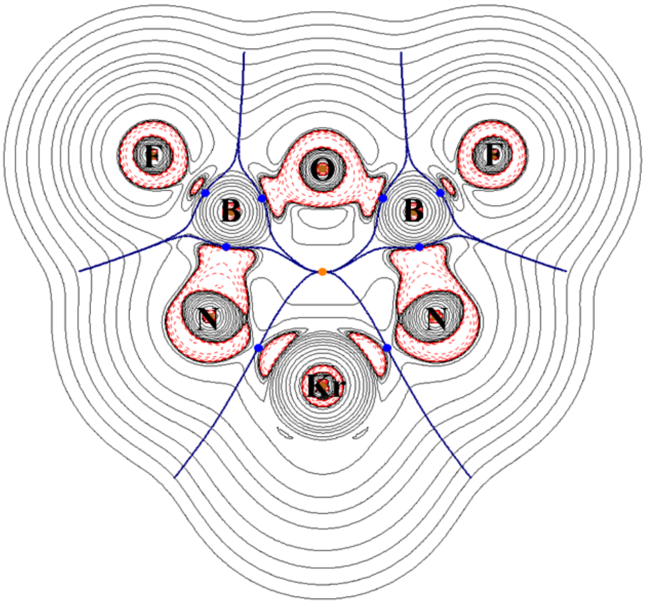

(a).

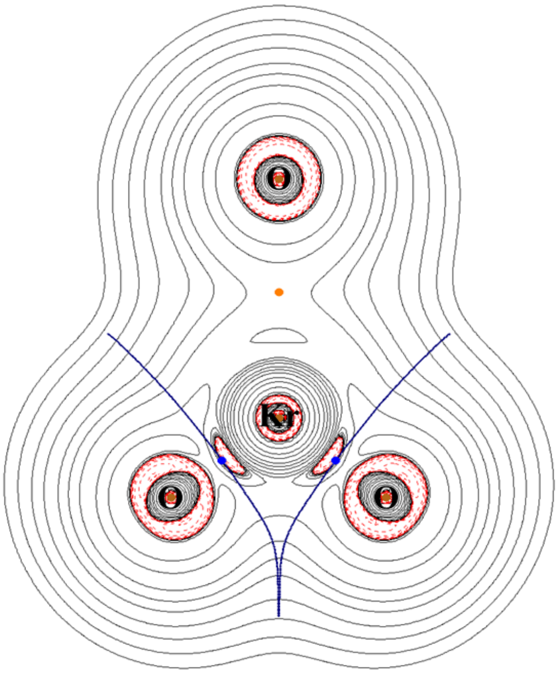

(b).

Figure S5. Contour plots of the calculated Laplace concentration of $KrO_3N_2B_2F_2$ on (a) the ring plane (b) the KrO_3 plane. The red contour lines are in regions of charge concentration and the black contour lines are in regions of charge depletion. The blue lines pass points of zero gradients, and the dots are bond critical points.

(a) 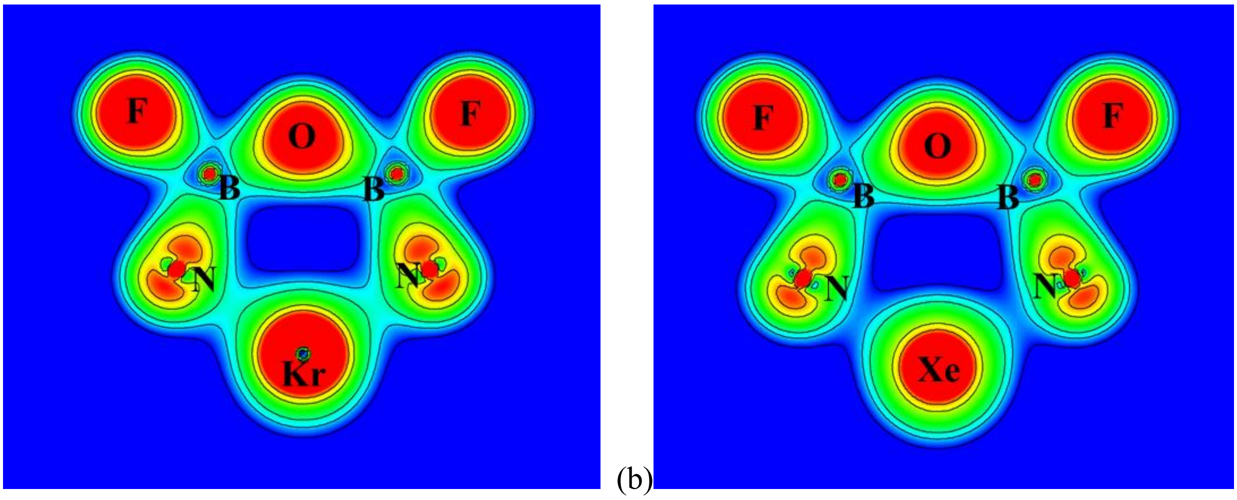 (b)

Figure S6. Contour plots of the calculated electron density of **P1.**

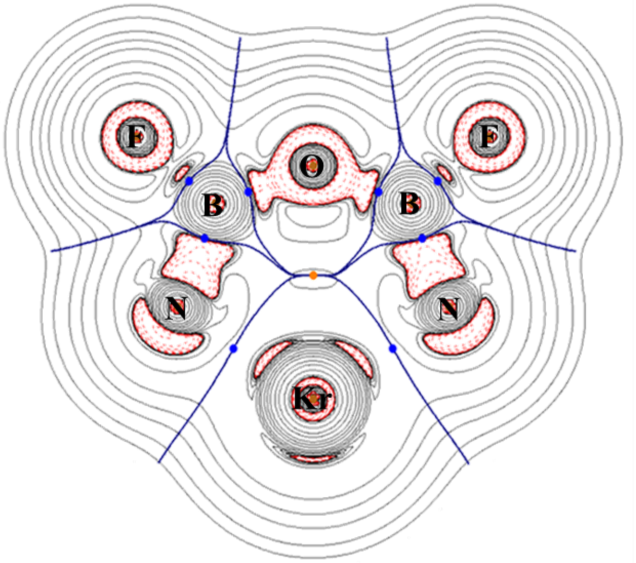

(a)

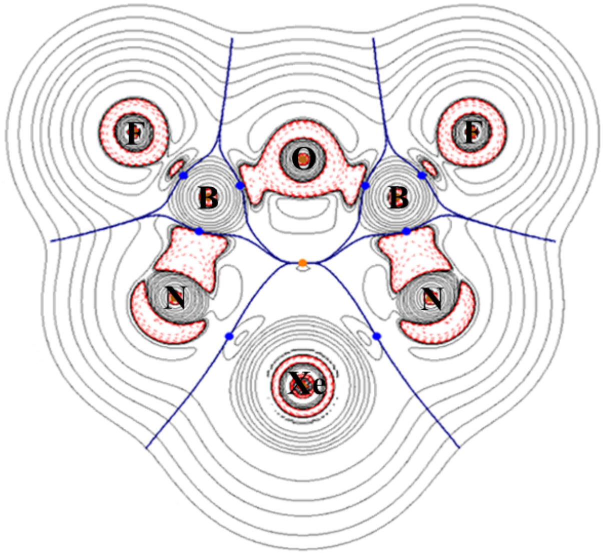

(b)

Figure S7. Contour plots of the calculated Laplace concentration of **P1**. The red contour lines are in regions of charge concentration and the black contour lines are in regions of charge depletion. The blue lines pass points of zero gradients, and the dots are bond critical points.

Table S1. Calculated structural parameters of **R**, $NgO_3N_2B_2F_2$ (Ng = Kr and Xe). The bond distances are in angstroms and bond angles in degrees.

	**MP2/aptz**	**B3LYP/aptz**	**M06-2X/aptz**
Ng = Kr			
R(Ng-N)	1.693	1.797	1.738
R(Ng-O)	1.588	1.645	1.616
R(O-B)	1.370	1.366	1.361
R(B-N)	1.461	1.454	1.454
R(B-F)	1.319	1.322	1.314
A(B-O-B)	126.1	129.9	128.6
A(O-B-N)	125.9	126.8	125.9
A(Ng-N-B)	112.2	109.8	111.2
A(N-Ng-N)	117.7	117.0	117.3
A(O-Ng-O)	106.9	106.8	107.1
Ng = Xe			
R(Ng-N)	1.847	1.912	1.869
R(Ng-O)	1.746	1.785	1.759
R(O-B)	1.375	1.370	1.366
R(B-N)	1.466	1.457	1.457
R(B-F)	1.320	1.323	1.315
A(B-O-B)	130.2	132.6	132.1
A(O-B-N)	126.9	127.2	126.4
A(Ng-N-B)	110.7	109.9	110.7
A(N-Ng-N)	114.5	113.1	113.6
A(O-Ng-O)	105.9	105.6	106.2

Table S2. The calculated structural parameters of **C1**, $NgO_2N_2B_2F_2$ (Ng = Kr and Xe). The bond distances are in angstroms and bond angles in degrees.

	MP2/aptz	B3LYP/aptz	M06-2X/aptz
Ng = Kr			
R(Ng-O)	1.623	1.703	1.672
R(Ng-N)	1.718	1.849	1.799
R(O-B)	1.374	1.368	1.365
R(B-N)	1.459	1.449	1.451
R(B-F)	1.322	1.326	1.318
A(B-O-B)	126.3	130.1	129.2
A(O-B-N)	125.2	126.8	126.0
A(Ng-N-B)	110.1	108.5	108.1
A(N-Ng-N)	114.7	113.6	114.4
Ng = Xe			
R(Ng-O)	1.770	1.819	1.794
R(Ng-N)	1.857	1.934	1.902
R(O-B)	1.377	1.372	1.368
R(B-N)	1.457	1.449	1.448
R(B-F)	1.325	1.328	1.332
A(B-O-B)	129.4	131.8	131.5
A(O-B-N)	126.3	127.0	126.6
A(Ng-N-B)	111.9	110.9	110.7
A(N-Ng-N)	110.5	109.6	110.3

Table S3. The calculated structure parameters of **P1**, $NgON_2B_2F_2$ (Ng = Kr and Xe). The bond distances are in angstroms and bond angles in degrees.

	**MP2/aptz**	**B3LYP/aptz**	**M06-2X/aptz**
Ng = Kr			
R(Ng-N)	1.885	2.130	2.013
R(O-B)	1.378	1.365	1.363
R(B-N)	1.442	1.427	1.435
R(B-F)	1.333	1.340	1.329
A(B-O-B)	130.9	137.6	135.9
A(O-B-N)	128.6	131.2	129.9
A(Ng-N-B)	105.1	104.2	104.4
A(N-Ng-N)	118.0	111.5	115.6
Ng = Xe			
R(Ng-N)	1.956	2.100	2.053
R(O-B)	1.385	1.373	1.369
R(B-N)	1.443	1.433	1.435
R(B-F)	1.334	1.339	1.330
A(B-O-B)	130.6	136.3	135.7
A(O-B-N)	128.1	130.3	129.7
A(Ng-N-B)	106.3	106.2	106.5
A(N-Ng-N)	112.2	110.7	111.9

Table S4. The calculated dissociation energies of R1 and the vertical singlet-triplet energy gaps (kcal/mol) of **R** at various theoretical levels.

	**C1 + O(S)**	**C1 + O(T)**	**P1+ O(S) + O(S)**	**P1+ O(T) + O(T)**	**S-T gap**
Ng = Kr					
MP2/aptz	77.5	11.4	148.9	16.7	95.9
B3LYP/aptz	59.5	-3.9	114.0	-12.8	65.1
M06-2X/aptz	57.7	-2.1	106.7	-12.6	82.2
CCSD(T)/aptz[a]	49.5	-1.3	93.9	-7.8	71.8
Ng = Xe					
MP2/aptz	98.4	32.3	206.1	73.9	103.6
B3LYP/aptz	81.0	17.6	169.0	42.1	63.7
M06-2X/aptz	77.5	17.9	155.4	36.1	91.2
CCSD(T)/aptz[a]	72.6	21.7	153.3	51.6	73.7

[a]Single-point calculation using B3LYP/aptz structures.

Table S5. Calculated dissociation barriers and reaction energies (kcal/mol) of R2 relatived to **R**.

	**Barrier (TS1)**	**Complex (C2)**	**Barrier (TS2)**	**NgO_2 + $ON_2B_2F_2$ (P2)**
Ng = Kr				
MP2/aptz	37.7	23.5	34.9	-106.0
B3LYP/aptz	15.5	10.1	15.4	-122.7
M06-2X/aptz	22.0	13.7	19.3	-121.1
CCSD(T)/aptz[a]	21.9	14.5	19.6	-116.5
Ng = Xe				
MP2/aptz	45.7	31.2	45.7	-63.9
B3LYP/aptz	26.3	18.5	24.7	-85.9
M06-2X/aptz	33.8	21.7	29.1	-86.0
CCSD(T)/aptz[a]	35.7	25.1	32.0	-74.1

[a]Single-point calculation using B3LYP/aptz structures.

Table S6. Calculated dissociation barriers and reaction energies (kcal/mol) of R3 relative to **R**.

	**Barrier (TS3)**	**$NgON_2B_2F_2$ (P1) + O_2**
Ng = Kr		
MP2/aptz[a]	90.5	-84.8
B3LYP/aptz	53.1	-97.5
M06-2X/aptz[a]	65.4	-96.6
CCSD(T)/aptz[a]	61.7	-92.6
Ng = Xe		
MP2/aptz[a]	103.1	-22.2
B3LYP/aptz	54.7	-42.6
M06-2X/aptz	71.9	-47.8
CCSD(T)/aptz[a]	71.4	-33.2

[a]Single-point calculation using B3LYP/aptz structures.

Table S7. Calculated dissociation barriers, reaction energies, and the vertical singlet-triplet energy gaps (kcal/mol) of **P1**.

	**Barrier (TS4)**	**Complex (C3)**	**Barrier (TS5)**	**Ng + (P2)**	**S-T gap**
Ng = Kr					
MP2/aptz	12.0	1.4	12.2	-160.7	42.8
B3LYP/aptz	4.9	0.4	4.9	-147.9	21.8
M06-2X/aptz	5.4	1.0	5.4	-152.2	39.9
CCSD(T)/aptz[a]	5.7	1.1	5.7	-150.8	19.4
Ng = Xe					
MP2/aptz	28.7	17.5	29.0	-128.6	55.6
B3LYP/aptz	13.7	9.5	16.2	-128.7	34.4
M06-2X/aptz	15.2	10.4	16.1	-132.7	48.6
CCSD(T)/aptz[a]	17.6	13.0	18.3	-126.1	37.3

[a]Single-point calculation using B3LYP/aptz structures.

Table S8. Electron density (ρ), the Laplacian of electron density ($\nabla^2\rho$) and the local energy density H(r_c) at the bond critical points (BCPs) in $NgO_3N_2B_2F_2$ using the electron density calculated at the B3LYP/aptz level.

$NgO_3N_2B_2F_2$	ρ(*r*)	∇^2ρ(*r*)	*H*(*r*)
Ng = Kr			
B–F	0.211	1.162	-0.172
B–N	0.196	0.233	-0.201
B–O	0.209	0.766	-0.190
Kr–N	0.195	0.069	-0.118
Kr–O	0.269	-0.011	-0.232
Ng = Xe			
B–F	0.211	1.157	-0.172
B–N	0.194	0.219	-0.199
B–O	0.206	0.753	-0.186
Xe–N	0.178	0.020	-0.117
Xe–O	0.217	0.215	-0.174

Table S9. Calculated cartesian coordinates (in Å) of R, $NgO_3N_2B_2F_2$ (Ng = Kr and Xe) at B3LYP/aptz level.

R

Ng = Kr

N	0.000000	1.531986	0.051717
N	0.000000	-1.531986	0.051717
O	-1.320903	0.000000	1.971390
O	1.320903	0.000000	1.971390
B	0.000000	1.236995	-1.371738
B	0.000000	-1.236995	-1.371738
F	0.000000	2.308990	-2.145274
F	0.000000	-2.308990	-2.145274
O	0.000000	0.000000	-1.950496
Kr	0.000000	0.000000	0.990833

Ng = Xe

N	0.000000	1.595740	-0.174249
N	0.000000	-1.595740	-0.174249
O	-1.421201	0.000000	1.958820
O	1.421201	0.000000	1.958820
B	0.000000	1.254979	-1.590621
B	0.000000	-1.254979	-1.590621
F	0.000000	2.294357	-2.408588
F	0.000000	-2.294357	-2.408588
O	0.000000	0.000000	-2.140951
Xe	0.000000	0.000000	0.879385

Table S10. Calculated cartesian coordinates (in Å) of the transition states at B3LYP/aptz level.

TS1

Ng = Kr

N	-1.022970	-1.751210	-0.162290
N	0.508832	1.409124	0.148777
O	2.321205	-0.136660	-1.247340
O	1.982038	-0.593700	1.401151
B	-2.162350	-0.951220	-0.077280
B	-0.927940	1.413661	0.024147
F	-3.419550	-1.332170	0.054088
F	-1.467110	2.623203	0.095328
O	-1.796790	0.371859	-0.171270
Kr	1.193852	-0.240800	-0.023470

Ng = Xe

N	-1.127490	-1.674970	-0.478640
N	0.122229	1.425151	0.355842
O	2.557750	0.470607	-0.828920
O	-2.077470	0.370517	-0.339090
B	-2.304370	-0.977570	-0.192580
B	-1.281360	1.424288	0.085474
F	-3.479000	-1.479670	0.139648
F	-1.915160	2.580233	0.230389
O	1.573383	-0.922040	1.473057
Xe	1.057105	-0.180420	-0.081030

TS2

Ng = Kr

N	-0.412780	1.337634	0.243292
N	1.162706	-0.762850	1.338630
O	1.911625	0.422276	-0.375010
O	-0.750960	-1.199990	-1.174200
B	0.984253	1.420206	-0.121470
B	2.011169	-0.816890	0.218878
F	1.420396	2.667037	-0.216580
F	2.783730	-1.786990	-0.218180
O	-2.817750	0.204089	-0.081650
Kr	-1.244640	-0.288100	0.149979

Ng = Xe

N	-0.074050	1.408114	0.172101
N	1.264133	-0.842750	1.235931
O	-0.661300	-1.232660	-1.259540
O	2.245143	0.391943	-0.233310
B	1.330121	1.443202	-0.101080
B	2.212995	-0.912640	0.206344
F	1.860951	2.646562	-0.257470
F	2.958375	-1.885490	-0.260040
O	-2.712420	0.511977	-0.255520
Xe	-1.118360	-0.200560	0.152999

TS3

Ng = Kr

N	-0.211060	1.472209	-0.184650
N	0.337625	-1.587170	-0.283710
O	-2.864260	0.366796	-0.430700
O	-1.743380	-0.525400	1.342569
B	1.223718	1.390239	0.050957
B	1.652508	-1.046820	-0.039140
F	1.810051	2.575661	0.117034
F	2.624249	-1.950270	-0.044550
O	1.982100	0.266314	0.167285
Kr	-0.949210	-0.205630	-0.168500

Note: TS3 for Ng = Kr needs a previously saved checkpoint file to converge to the correct state. At this geometry, there seem to be several low-lying singlet states that may cross each other. The authors can provide the checkpoint file upon request.

Ng = Xe

N	0.036610	1.537887	-0.105480
N	0.501241	-1.611690	-0.214370
O	2.190836	0.232223	0.130398
O	-2.689280	0.535000	-0.175990
B	1.474474	1.391724	0.067638
B	1.823923	-1.085470	-0.004200
F	2.134385	2.537571	0.159698
F	2.793092	-1.995280	0.038256
O	-1.675340	-0.504100	1.457665
Xe	-0.874330	-0.148150	-0.206600

TS4

Ng = Kr

N	0.880398	1.488710	-0.164510
N	-0.862700	-1.798460	-0.311860
B	-0.523900	1.407796	0.010911
B	-1.866600	-0.873570	-0.006030
F	-1.060740	2.636149	-0.084590
F	-3.162390	-1.161390	0.041389
O	-1.379890	0.370184	0.276044
Kr	1.690999	-0.464920	0.041405

Ng = Xe

N	0.404977	1.580454	-0.224950
N	-1.094290	-1.790910	-0.356870
B	-0.974010	1.381963	0.021916
B	-2.161430	-0.967820	0.002150
F	-1.687260	2.513082	-0.103640
F	-3.449760	-1.285960	0.016570
O	-1.680290	0.253036	0.397205
Xe	1.484775	-0.253070	0.028860

TS5

Ng = Kr

N	1.170966	-0.875640	1.359628
N	-0.685820	1.407568	0.345618
B	1.789980	-0.826220	0.096170
B	0.658737	1.326740	-0.110520
F	2.636961	-1.731020	-0.376930
F	1.247066	2.524521	0.002379
O	1.396955	0.271861	-0.627960
Kr	-1.715880	-0.431730	-0.096400

Ng = Xe

N	1.515627	-0.847640	1.359227
N	-0.277200	1.495686	0.312977
B	2.130665	-0.894720	0.098480
B	1.066196	1.320684	-0.107110
F	2.930881	-1.834570	-0.384820
F	1.774994	2.452734	-0.009170
O	1.737359	0.203721	-0.624100
Xe	-1.498240	-0.256660	-0.057850

Table S11. Calculated cartesian coordinates (in Å) of the complexes at B3LYP/aptz level.

C1

Ng = Kr

N	-0.348076	-0.202874	1.546728
N	-0.348076	-0.202874	-1.546728
O	1.378100	-1.954463	0.000000
B	-0.069787	1.185556	1.240385
B	-0.069787	1.185556	-1.240385
F	-0.069787	1.991712	2.293632
F	-0.069787	1.991712	-2.293632
O	0.134936	1.724942	0.000000
Kr	-0.146589	-1.195277	0.000000

Ng = Xe

N	0.274497	0.041077	1.579808
N	0.274497	0.041077	-1.579808
O	-1.547760	-1.804483	0.000000
B	0.095474	1.441237	1.252448
B	0.095474	1.441237	-1.252448
F	0.095474	2.273110	2.287097
F	0.095474	2.273110	-2.287097
O	-0.031997	1.986186	0.000000
Xe	0.113367	-1.062167	0.000000

C2

Ng = Kr

N	-0.432630	1.356200	-0.203110
N	1.180112	-0.921390	1.271651
O	1.920378	0.445459	0.342989
O	-0.924140	-1.328590	-1.079390
B	0.982237	1.414071	-0.065920
B	2.063646	-0.978130	0.241482
F	1.533745	2.585040	-0.348750
F	2.820003	-1.668560	-0.559900
O	-2.837480	0.245940	0.030448
Kr	-1.247660	-0.232620	0.151883

Ng = Xe

N	-1.290330	-0.979280	-1.147530
N	0.075233	1.394584	0.253839
O	2.740125	0.555791	0.148138
O	-2.166810	0.383567	-0.452510
B	-2.280820	-1.027830	-0.218380
B	-1.316960	1.410913	0.073599
F	-3.085180	-1.689790	0.555721
F	-2.006840	2.498799	0.381490
O	0.872422	-1.355710	1.175842
Xe	1.125126	-0.162460	-0.156060

C3

Ng = Kr

N	2.102924	-0.688208	1.423472
N	-0.862234	1.388065	-0.054995
B	2.368585	-0.682149	0.089627
B	0.496430	1.002708	-0.029900
F	3.267309	-0.914263	-0.837963
F	1.333711	2.046883	0.004796
O	1.004304	-0.297807	-0.102708
Kr	-2.012597	-0.397581	-0.043273

Ng = Xe

N	-0.409084	1.460732	-0.00685
N	2.393419	-0.842436	1.386994
B	0.9321	1.032833	-0.00978
B	2.737291	-0.728168	0.076791
F	1.841153	2.014993	0.05914
F	3.680818	-0.904889	-0.81896
O	1.393455	-0.291581	-0.14258
Xe	-1.723753	-0.250179	-0.03735

Table S12. Calculated cartesian coordinates (in Å) of P1 and P2 at B3LYP/aptz level.

P1

Ng = Kr

N	-0.000082	0.287073	1.760617
N	-0.000082	0.287073	-1.760617
B	-0.000634	-1.053818	1.272737
B	-0.000634	-1.053818	-1.272737
F	-0.000082	-1.921259	2.294208
F	-0.000082	-1.921259	-2.294208
O	-0.000631	-1.547220	0.000000
Kr	0.000389	1.485544	0.000000

Ng = Xe

N	1.726866	0.041252	0.000000
N	-1.726865	0.041252	0.000000
B	1.273728	-1.317922	0.000000
B	-1.273727	-1.317922	0.000000
F	2.284665	-2.195755	0.000000
F	-2.284666	-2.195753	0.000000
O	0.000000	-1.830109	0.000000
Xe	0.000000	1.236410	0.000000

P2

N	-0.000651	1.331800	0.629979
N	-0.000651	1.331800	-0.629979
B	0.000045	-0.132861	1.079178
B	0.000045	-0.132861	-1.079178
F	0.000045	-0.523205	2.322259
F	0.000045	-0.523205	-2.322259
O	0.000980	-0.987363	0.000000
